# Analysis of the human cytomegalovirus genomic region from UL146 through UL147A reveals sequence hypervariability, genotypic stability, and overlapping transcripts

**DOI:** 10.1186/1743-422X-3-4

**Published:** 2006-01-12

**Authors:** Nell S Lurain, Andrea M Fox, Heather M Lichy, Sangeeta M Bhorade, Carl F Ware, Diana D Huang, Sau-Ping Kwan, Edward R Garrity, Sunwen Chou

**Affiliations:** 1 Department of Immunology/Microbiology, Rush University Medical Center, Chicago, IL, USA; 2 Medical and Research Services, VA Medical Center, Portland, OR, USA; 3 Division of Infectious Diseases, Oregon Health & Science University, Portland, OR, USA; 4 Division of Pulmonary and Critical Care, Loyola University Medical Center, Maywood, IL, USA; 5 Division of Molecular Immunology, La Jolla Institute for Allergy and Immunology, San Diego, CA, USA

## Abstract

**Background:**

Although the sequence of the human cytomegalovirus (HCMV) genome is generally conserved among unrelated clinical strains, some open reading frames (ORFs) are highly variable. UL146 and UL147, which encode CXC chemokine homologues are among these variable ORFs.

**Results:**

The region of the HCMV genome from UL146 through UL147A was analyzed in clinical strains for sequence variability, genotypic stability, and transcriptional expression. The UL146 sequences in clinical strains from two geographically distant sites were assigned to 12 sequence groups that differ by over 60% at the amino acid level. The same groups were generated by sequences from the UL146-UL147 intergenic region and the UL147 ORF. In contrast to the high level of sequence variability among unrelated clinical strains, the sequences of UL146 through UL147A from isolates of the same strain were highly stable after repeated passage both in vitro and in vivo. Riboprobes homologous to these ORFs detected multiple overlapping transcripts differing in temporal expression. UL146 sequences are present only on the largest transcript, which also contains all of the downstream ORFs including UL148 and UL132. The sizes and hybridization patterns of the transcripts are consistent with a common 3'-terminus downstream of the UL132 ORF. Early-late expression of the transcripts associated with UL146 and UL147 is compatible with the potential role of CXC chemokines in pathogenesis associated with viral replication.

**Conclusion:**

Clinical isolates from two different geographic sites cluster in the same groups based on the hypervariability of the UL146, UL147, or the intergenic sequences, which provides strong evidence for linkage and no evidence for interstrain recombination within this region. The sequence of individual strains was absolutely stable in vitro and in vivo, which indicates that sequence drift is not a mechanism for the observed sequence hypervariability. There is also no evidence of transcriptional splicing, although multiple overlapping transcripts extending into the adjacent UL148 and UL132 open reading frames were detected using gene-specific probes.

## Background

Human cytomegalovirus (HCMV) has a double-stranded linear DNA genome of approximately 235 kbp in length making it the largest of the human herpesviruses. Analysis of the complete genome of several strains predicts over 160 open reading frames (ORFs) [1-3]. The overall nucleotide sequence of strains isolated from unrelated sources is relatively conserved, however, the sequences of specific ORFs can be highly variable. Sequence variation was initially described in the glycoprotein B (gB) gene of clinical HCMV strains [4]. The discovery of a genomic region in the Toledo strain that had been deleted from the prototype laboratory strain AD169 [5], added a new set of previously unrecognized open reading frames (ORFs). Sequence comparisons of specific ORFs in this region as well as in the remainder of the genome of HCMV clinical isolates have revealed a surprisingly high level of variability. These ORFs include RL6, RL12, UL4, UL18, UL55 (gB), UL73 (gN), UL74 (gO), UL139, UL144, and UL146 [2,4,6-14]. The variability appears not to be randomly generated but usually occurs as a limited number of distinct sequence groups. The consensus nucleotide sequences of the groups may vary by as much as 50% or more depending on the ORF. In many cases the majority of nucleotide changes are non-synonymous, which results in similar variability for the predicted amino acid sequences.

There is relatively little evidence for linkage among these hypervariable genes. Variant groups of gN and gO, which are encoded by adjacent ORFs UL73 and UL74, appear to be strongly linked [15,16], although they are not found in the same glycoprotein complex. In contrast gO and gL whose products are components of the same glycoprotein complex are found in different genetic combinations in unrelated HCMV strains [17]. Attempts to establish linkage of gB sequence groups with several other hypervariable ORFs have generally produced negative results [7,8,13,16].

Several of the encoded products of the hypervariable ORFs in HCMV have predicted immunomodulatory function. Of particular interest is the UL146 ORF, which encodes a C-X-C chemokine homologue [18,19]. The UL146 product has functions associated with α-chemokines including induction of chemotaxis, calcium flux, and neutrophil degranulation [18,20]. The product also appears to be required for infection of neutrophils [21]. Despite these established functions, UL146 is among the most variable of the HCMV ORFs. Although the variability occurs throughout the nucleotide sequence [2], phylogenetic analyses have shown that the UL146 sequences of clinically unrelated patients cluster in defined sequence groups [2,22].

The adjacent UL147 ORF, which is also variable, encodes a second C-X-C chemokine homologue, although no chemokine-associated activity has been reported [19]. The UL147A ORF begins only two nucleotides downstream of the UL147 coding sequence, but no function has been assigned to the predicted product [2,3,23,24].

In the current study, we characterized the UL146 sequences, the neighboring ORFs UL147 and UL147A, and the intergenic regions of a large number of HCMV clinical isolates focusing on the following characteristics of this genomic region: 1) the variability and sequence stability of HCMV clinical strains during long-term replication both in vitro and in vivo; 2) the potential linkage of the UL146, UL147, and UL147A ORFs; and 3) the transcriptional pattern associated with these ORFs.

## Results

### Sequence variability of the UL146 ORF

Phylogenetic analysis was performed on a total of 50 UL146 sequences. Of these, 48 were obtained from clinical HCMV strains isolated from different patients at the two different sites: 29 strains from Chicago and 19 strains from Portland. The highly-characterized strains Towne and Toledo were also included in the phylogenetic analysis. Among these strains the length of the ORF ranges from 342 to 375 nucleotides encoding predicted proteins of 114 to 125 amino acids. The nucleotide variability is as high as 58% between strains such as CH-14 and PT-18 (Figure 2). Despite this variability the strains can be placed into 12 discrete sequence groups differing by at least 10%. The amino acid sequences generate the same groups with the overall percentage of variability greater than 60% (Figure 2). These have been numbered in Figure 2 based on homology with groups defined by Dolan et al. [2], who reported a total of 14 sequence groups. There were no strains homologous to their groups 4 and 6. In some cases, strains from Chicago and Portland have identical UL146 nucleotide sequences. For example the sequence of PT-18 (Chicago) is identical to that of C194 (Portland) and BI-5 (Chicago) is identical to C954 (Portland).

**Figure 1:**
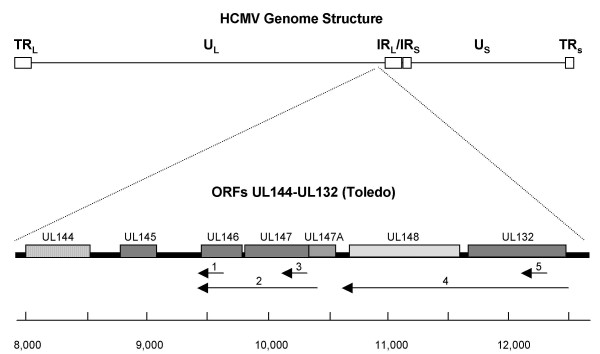
**Map of HCMV UL144 through UL132 open reading frames**. Upper map shows the general structure of the complete HCMV genome. Lower map is an expansion of the region of interest at the indicated position on the HCMV genome. U_L_, unique long; U_S_, unique short; IR_L_, internal repeat long; IR_S_, internal repeat short; TR_L_, terminal repeat long; TR_S_, terminal repeat short. Numbered arrows indicate riboprobe sequences. Nucleotide numbers below the map are based on the Toledo sequence (GenBank accession number U33331).

**Figure 2:**
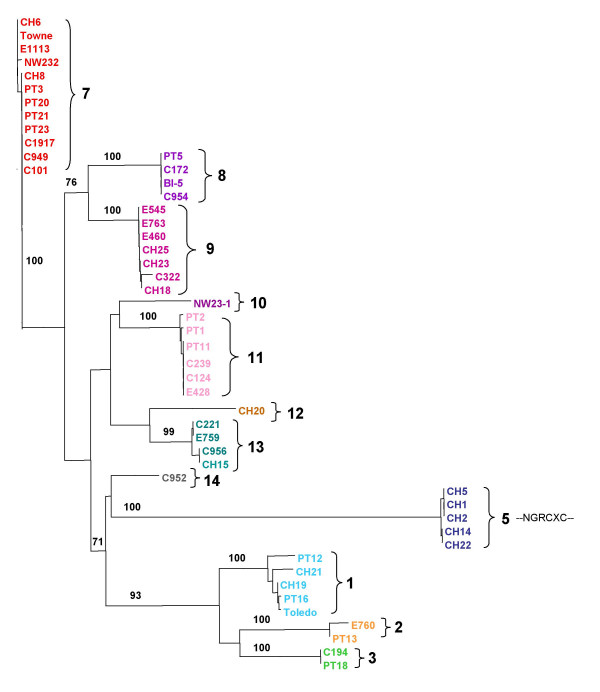
**UL146 phylogenetic analysis**. UL146 amino acid sequences from 48 clinical strains, plus Towne and Toledo. Group designations correspond to those of Dolan et al. [2].

The CXC chemokine motif is conserved in all strains, and the majority of the UL146 sequences have an adjacent ELR motif as well. The ELR residues have been shown to be required for chemokine function and binding and activation of the receptors CXCR1 and CXCR2 [25-27]. The variable X residue of the CXC motif in our strains is most often proline, although arginine, threonine, and lysine are also found in that order of frequency. Five strains, CH-1, CH-2, CH-5, CH-14, and CH-22 from Chicago have the ELR motif replaced by the variant NGR sequence (Figure 2, Group 5), which has been described in other studies [2,9,10,22]. In addition, the complete UL146 nucleotide sequences of these 5 strains in Group 5 are either identical or differ by no more than a single nucleotide, and the X residue in the CXC motif is consistently threonine. Besides the cysteine residues in the CXC motif, each of the 50 strains has two additional cysteine residues, which occur at approximately the same positions in the individual strain sequences. These residues are homologous to those of the prototype CXC chemokine, IL-8, which form disulfide bonds with the cysteines of the CXC motif [25,28]. Predicted N-linked glycosylation sites are not conserved among the UL146 sequence groups. Group 1 containing the Toledo strain has three predicted sites, one of which lies within a putative signal sequence [2]. Other groups have only single sites (Group 8) or lack sites altogether (Group 7).

The hypervariability of the UL146 ORF led to an analysis of the sequence components downstream of the UL146 stop codon. A subset of 32 strains was selected for this analysis: 17 from Chicago and 13 from Portland plus Towne and Toledo. This included the intergenic region between UL146 and UL147 plus each of the downstream ORFs, UL147 and UL147A. The sequence groups that were defined by the original set of 50 strains are all represented except Group 3.

### Sequence variability of the intergenic region between UL146 and UL147

The intergenic region between UL146 and UL147 showed a very high degree of variability. This is the result of not only nucleotide differences, but also the length of this non-coding region, which ranges from 43 to 214 bp depending on the strain. However, within sequence groups defined by UL146, the associated intergenic nucleotide sequences and sequence lengths are either identical or differ by no more than 2 nucleotides, and all are AT rich.

**Figure 3:**
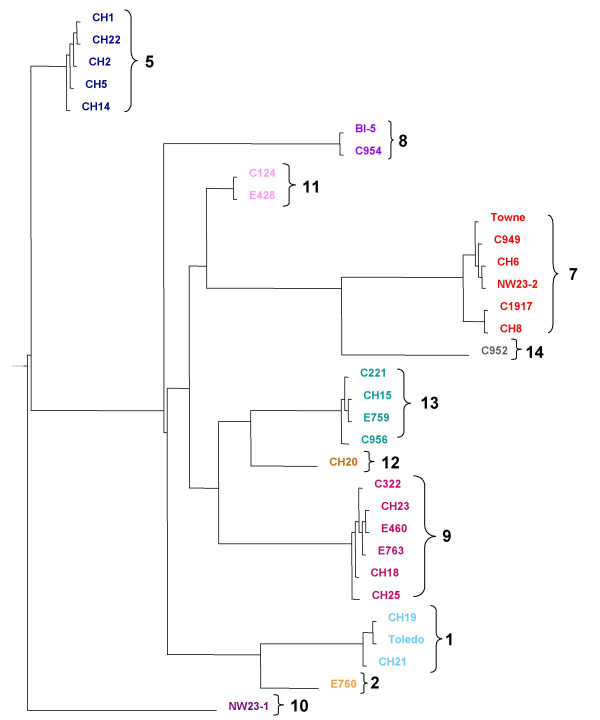
**UL146-147 intergenic region phylogenetic analysis**. Dendrogram of intergenic nucleotide sequences from 32 strains. Group designations are the same as those in Figure 2.

A dendrogram of the intergenic sequence groups (Figure 3) shows the same relationships of the strains within the groups as those determined by phylogenetic analysis of the UL146 sequence. For example all Group 7 strains based on intergenic sequences in Figure 3 are found in Group 7 strains based on UL146 in Figure 2. The longest intergenic sequence was found exclusively associated with sequences that have the aberrant NGRCXC chemokine motif, which are all in Group 5. These results indicate that the UL146 sequences are consistently linked to specific intergenic sequences.

### Sequence variability of UL147

**Figure 4:**
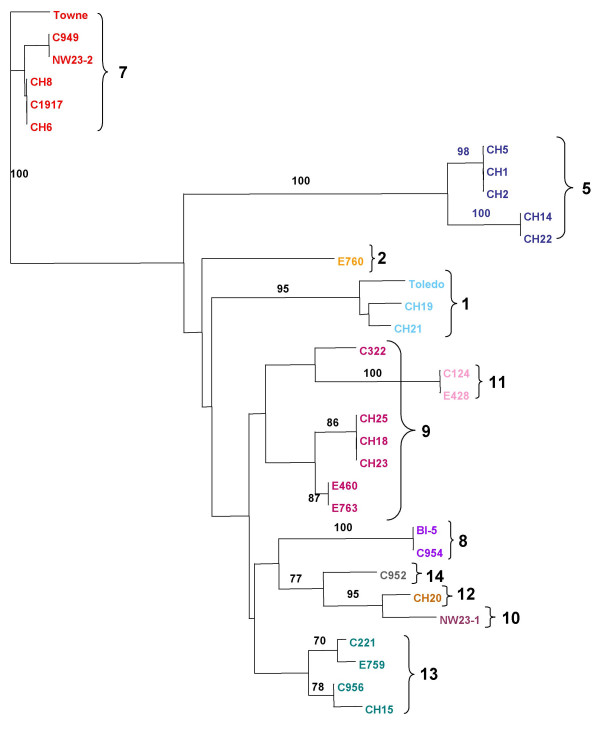
**UL147 phylogenetic analysis**. UL147 amino acid sequences from 32 strains. Group designations are the same as those in Figure 2.

Alignment of the UL147 ORF sequences in the 32 isolates showed that the 5' half (basepairs 1–216) varied by as much as 22% at the nucleotide level with a corresponding amino acid variability of 26%. By comparison the 3' half of the ORF (basepairs 217–480) showed only 3–4% nucleotide and amino acid sequence variability. The overall variability of the UL147 ORF is approximately 15%. The length ranges from 474 to 480 nucleotides, which translates into a difference of only 2 amino acids among unrelated strains. Several isolates have 2 adjacent methionine codons at the predicted start site, which leads to ambiguity in determining the initiation codon. There is a completely conserved DXRCXC chemokine motif where the first X represents either an arginine or lysine residue and the second X is always an arginine residue in the strains that we analyzed. Phylogenetic analysis based on UL147 alone (Figure 4), again showed sequence groups very similar to those determined by UL146 sequences, which suggests that the linkage observed for UL146 and the intergenic region can be extended to include UL147. There are no N-linked glycosylation sites encoded by any of the 32 UL147 amino acid sequences.

### Sequence variability of UL147A

The predicted initiation codon of the UL147A ORF is invariably 2 nucleotides downstream of the UL147 stop codon, which shifts the reading frame from that of UL147. The UL147A ORF is highly conserved among all strains with a maximum of 6–7% variability in the nucleotide and amino acid sequences. The length of the ORF is invariably 228 nucleotides. Sequence groups are not distinguishable, because of the high level of conservation. There are no N-linked glycosylation sites.

Table 1 summarizes the characteristics of the 3 ORFs UL146, UL147, and UL147A and the UL146-147 intergenic region based on the analysis of the 32 isolates. All components except UL147A have different nucleotide lengths. In general the non-synonymous nucleotide substitutions are reflected by a slightly greater variability at the amino acid level. Despite this hypervariability, isolates BI-5 from Chicago and C954 from Portland have identical sequences for the entire region UL146 through UL147A.

**Table 1:** Characteristics of open reading frames UL146, UL147, and UL147A.

Open reading frame	Number of nucleotides	Number of amino acids	Maximum nucleotide variability	Maximum amino acid variability
UL146	342–375	114–125	58%	61%
Intergenic region	43–214	-----	-----	-----
UL147	471–477	157–159	13%	15%
UL147A	228	76	7%	8%

### UL144 sequence groups

Previous work with the UL144 ORF, which is a TNF receptor homologue [29], showed that there is sequence variability of up to 20% and the sequences are distributed among three major groups one of which can be divided into 3 subgroups [6,7,30,31]. A limited number of UL144 sequences from the Chicago strains were compared using the previously published UL144 group designations [7] to determine whether there is any linkage between UL144 groups and those defined by the UL146-UL147A region. There was no evidence of linkage as demonstrated by several strains that grouped together based on similar UL146-UL147A sequences but differed in their UL144 sequences. For example, NW23-2, PT3, and PT20, are all in the same UL146 group (Group 7, Figure 2), but they represent UL144 groups 2, 3, and 1A respectively [7].

### Sequence stability of the UL146 ORF in virus isolates passaged in vitro

A possible mechanism for generating the UL146 hypervariability could be gradual drift during long-term virus replication. To address this possibility by in vitro methods, 11 isolates from Portland and 2 from Chicago were each passaged a minimum of 10 times and as many as 47 times in cell culture (Table 2). The phenotype of the isolates changed from cell-associated to extracellular as a result of these multiple passages. Despite the change in phenotype, all strains in Table 2 with the same designation have identical nucleotide sequences. The results demonstrate that passage of the same strains in cell culture over several months produced no detectable changes in the nucleotide sequence of the UL146 ORF. This is particularly evident in comparing the nucleotide sequences of the low passage versus high passage isolates from subjects N, P, G, and T, which are identical.

**Table 2:** Serial isolates or passages from the same or related solid organ transplant recipients.^a^

Isolate number	Subjects	Donor/Recipient	Specimen type	Days post transplant	Passage	Strain	Relationship among strains
C307	N	D+R-	urine	68	3	N1	Unchanged UL146 sequences in isolates from individual subjects (N, P, G, F, T) after multiple passages in cell culture
C322	N		urine	68	13	N1	
							
X121	P	D+R+	urine	74	0	P1	
C336	P		urine	74	6	P1	
C956	P		urine	74	32	P1	
							
C2571	G	D+R+	urine	54	3	G1	
C952	G		urine	54	24	G1	
							
C194	F	D+R+	urine	101	5	F1	
C875	F		urine	101	9	F1	
							
C184	T	D+R-	urine	54	3	T1	
C221	T		urine	54	22	T1	

C108	R1	D+R+	blood	108	30	W1	R1 and R2 received kidneys from same donor
C246	R1		blood	108	47	W1	
C124	R1		urine	234	5	W1	
C345	R1		urine	234	29	W1	
							
C237	R1		urine	92	7	W3	Two different strains W1 and W3 shed by subject R1
X163	R1		urine	150	0	W3	
C101	R1		urine	234	5	W3	
C201	R1		urine	234	29	W3	
							
X199	R2	D+R-	urine	171	0	W1	Single strain W1 in these specimens from subject R2, but other specimens contained both W1 and W3 from the common donor
X89	R2		urine	255	0	W1	
C239	R2		urine	2 years	15	W1	

C3	L	D+R+	urine	111	12	L1	L and Z received kidneys from same donor
							
C1917	Z	D+R-	urine	216	9	L1	
C198	Z		urine	2.6 years	4	L1	

X162	C	D+R+	urine	55	0	C1	C and Y received kidneys from same donor.
C118	C		urine	55	4	C1	
C954	C		urine	55	16	C1	
							
C69	Y	D+R-	urine	21	4	C1	
C172	Y		urine	21	8	C1	

CH1-A	CH1	D+R-	BAL	1.1 years	>10	CH1-1	CH1 and CH2 received lungs from same donor.
CH1-B	CH1		BAL	4.5 years	8	CH1-1	
							
CH2-A	CH2	D+R-	BAL	150	>10	CH1-1	

NW23-A	NW23	D+R+	colon	1 year	5	NW23-1	Strain NW23-1 phylogenetically different from NW23-2. Specimens NW23-B and NW23-C collected 5 1/2 years apart.
NW23-B	NW23		BAL	1.6 years	5	NW23-2	
NW23-C	NW23		BAL	7 years	5	NW23-2	

^a^Specimens designated with C, CH, or NW were cultured. Specimens designated with X were analyzed directly.

All strains with the same strain designation had identical nucleotide sequences in UL146. Overall identity of strains from recipient pairs C/Y, L/Z, R1/R2, and CH1/CH2 with common donors was documented by Southern blot analysis [32, 48].

### Sequence stability and analysis of the UL146 ORF in virus isolates passaged in vivo

Because cell culture represents only one cell type and contains no cells of the immune system, the effect of long-term infection on UL146 variability in vivo was investigated. Multiple isolates were collected from some of the patients over a period of several months to several years at both the Chicago and Portland sites. The UL146 nucleotide sequence of each individual strain was found to be identical for all isolates from the same patient for periods of up to several years, as shown by isolate pairs NW23-B/C, CH1-A/B, X199/C239 and C1917/C198 in Table 2.

There were 3 pairs of kidney transplant recipients from Portland and 1 pair of lung transplant recipients from Chicago, for which both recipients received organs from the same donor. These are patients R1 and R2, L and Z, C and Y, CH-1 and CH-2 (Table 2). All donors were HCMV seropositive (D+) and some were documented to have transmitted one or more strains of CMV to multiple recipients. Isolates were obtained over periods of up to 5 years post-transplant. All isolates from recipients having the same donor had identical nucleotide sequences. Thus, passage of the same strain in different hosts over long periods of time did not alter the UL146 sequence. Some of these strains were also subjected to extensive passage in cell culture and still retained absolute sequence stability. Of note in this regard is the stability of strain W1 from isolate C246, which represents cell culture passage 47 of a blood isolate from 108 days post-transplant.

In some cases there was evidence that patients were infected with more than one strain. However, the integrity of individual strains within the same patient was maintained. For example, Chicago isolates NW23-A and NW23-B are from the same patient. The UL146 sequence of the earlier isolate, NW23-A (Group 10), from a colon biopsy differs significantly from that of NW23-B (Group 7), an isolate obtained 5 months later from a bronchoaveolar lavage (BAL). A third isolate, NW23-C also from a BAL obtained more than 5 years later is identical in sequence to NW23-B. The recipient was HCMV seropositive and received an organ from a seropositive donor (D+/R+), therefore, the recipient was infected with two different strains (NW23-1 and NW23-2) from two unrelated sources. The original isolate likely represents reactivation of the endogenous HCMV strain present pre-transplant, and the later isolates were derived from the donor strain. Kidney transplant recipients R1 and R2 had 2 strains in common, although recipient R2 shed one of the strains only intermittently (Table 2) [32]. Identical sequences for each strain were found in isolates from different compartments (blood and urine) and isolates obtained at different times post-transplant.

### Sequence stability of UL147 and UL147A

To extend the analysis of strain stability, the UL147 and UL147A ORFs were sequenced in clinically related isolates obtained at different time points as much as several years apart. In a total of 8 different cases, all isolates from the same patient had identical UL147 and UL147A sequences. Thus, the same sequence stability observed for the UL146 ORF appears to apply to these ORFs as well.

### Transcriptional profile

**Figure 5:**
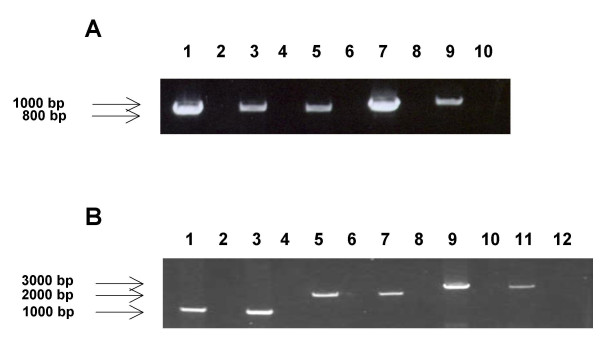
**RT-PCR amplification**. (A) RT-PCR products containing UL146 through UL147A orfs. Lane 1 NW23-1; Lane 2 NW23-1 no RT control. Lane 3 CH15; Lane 4 CH15 no RT control. Lane 5 CH25. Lane 6 CH25 no RT control. Lane 7 CH22; Lane 8 CH 22 no RT control. Lane 9 CH-14. Lane 10 no RT control. (B) RT-PCR products containing portions of region UL146 through UL132 from strains CH21 and CH23. Lane 1 CH21 UL146-UL147A; Lane 2 no RT control. Lane 3 CH23 UL146-UL147A; Lane 4 no RT control. Lane 5 CH21 UL146-UL148; Lane 6 no RT control. Lane 7 CH23 UL146-UL148; Lane 8 no RT control; Lane 9 CH21 UL146-UL132; Lane 10 no RT control; Lane 11 CH23 UL146-132; Lane 12 no RT control.

The transcriptional pattern associated with this group of ORFs was analyzed to further characterize their relationship from the standpoint of potential splicing and temporal expression. RNA was extracted from virus-infected cells at 10–14 days post-infection. Figure 5A shows representative RT-PCR products generated by the forward primer upstream of UL146 and the reverse primer downstream of UL147A. A total of 15 UL146-UL147A RT-PCR products amplified from unrelated Chicago strains were sequenced. These strains represent different UL146 sequence groups. In each case the sequence of the RT-PCR product was identical to the sequence of the PCR product derived from the viral genomic DNA. The differences in the sizes of the products reflect differences in the lengths of the intergenic regions. These results indicated that there is a single transcript that contains all three ORFs with no evidence of splicing. The RT-PCR analysis was extended using the same forward primer upstream of UL146 with primers downstream of UL148 and UL132. Figure 5B shows cDNA products from strains CH-21 (Group 1) and CH23 (Group 9) amplified by each of the three sets of primers. The sizes correspond to the products predicted by the genomic sequence. Sequencing of the UL146-UL148 and UL146-UL132 products again showed no evidence of splicing. When a conserved forward primer upstream of UL145 was used with the reverse primer downstream of UL147A, no cDNA product was obtained indicating that the UL145 ORF is not on the same transcript that contains UL146, UL147, and UL147A.

**Figure 6:**
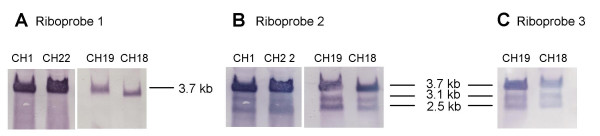
**Northern analysis of total transcripts from UL146 through UL132**. Transcriptional pattern associated with UL146 through UL132 from different HCMV clinical strains. (A) Total RNA extracted from cells infected with designated strains and hybridized with UL146 CH22-specific riboprobe number 1 (See Figure 1). (B) Same blot as in (A), but rehybridized with riboprobe number 2 (Figure 1) containing UL146 through UL147A orfs derived from strain CH22. (C) Total RNA extracted from cells infected with designated strains and hybridized with UL147 CH19-specific riboprobe number 3 (Figure 1).

Northern analysis was performed with 5 different riboprobes to determine the number and sizes of the transcripts containing these ORFs (Figure 1). At 7–10 days post-inoculation, RNA was extracted from cells infected with strains CH-1, CH-22, CH-18, and CH-19, which represent three different UL146 sequence groups (Groups 5, 9, and 1). Both CH-1 and CH-22 belong to the Group 5 strains that encode the NGRCXC motif (Figure 2). The extracted RNA was hybridized with riboprobes 1, 2, or 3 (Figure 1). Ribroprobe 1 was designed to be specifically antisense to the variable UL146 coding sequences of CH-22, although it contains approximately 80 basepairs of the upstream noncoding sequences, which are conserved among all of the strains. The probe hybridizes with a single transcript (Figure 6A). The hybridization is strongest with the homologous RNA from strains CH-1 and CH-22 (Group 5) and less intense with CH-18 (Group 9) and CH-19 (Group 1) RNA samples, which have much lower sequence homology. The completely homologous RNA bands (CH1 and CH2) are approximately 3.7 kb in size. The bands detected for CH19 and CH18, which have limited homology to the probe, are slightly smaller as predicted by the differences in the length of the UL146-UL147 intergenic region. Reprobing the same northern blot with riboprobe 2, which is antisense to the three ORFs (UL146, UL147, UL147A), shows 2 additional transcripts of approximately 3.1 and 2.5 kb in length (Figure 6B). Riboprobe 3, which contains only the relatively conserved 3-prime UL147 sequences, detects the same 3 transcripts (Figure 6C). These results indicate that UL146 sequences are present only on the largest transcript, which also contains UL147 sequences. The smaller transcripts contain UL147 sequences but have no sequences in common with UL146.

**Figure 7:**
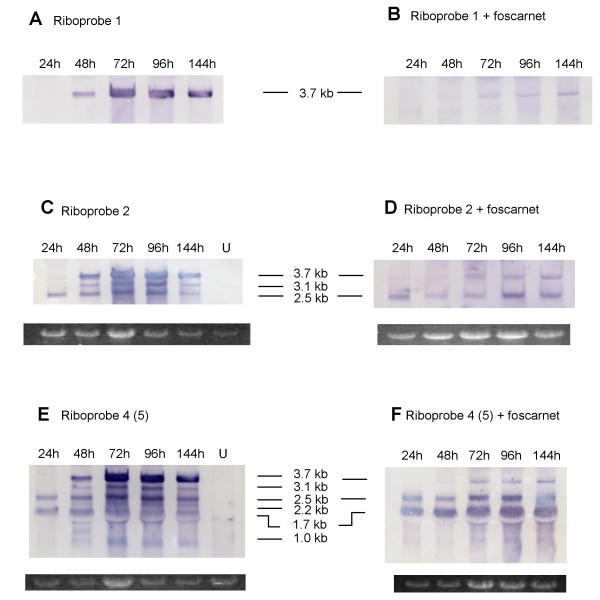
**Northern analysis of temporal transcriptional pattern of UL146 through UL132**. Total RNA was extracted at the indicated time points post-inoculation from cells infected with strain CH2 and hybridized with riboprobe number 1 derived from strain CH22 in the absence (A) or presence (B) of 400 μM foscarnet. (C) and (D) same as (A) and (B) but hybridized with riboprobe number 2. (E) and (F) same as (C) and (D) but hybridized with riboprobe 4. Lanes labeled U contain RNA from uninfected cells. Ribroprobe 5 data are not shown but are identical to ribroprobe 4 data as indicated by parentheses. Agarose gels showing 28S ribosomal RNA for loading control below (C) through (F).

To determine the temporal expression of the transcripts, RNA produced in the presence or absence of the DNA replication inhibitor, foscarnet (400 μM), was obtained for northern analysis at different time points post-inoculation. Using riboprobe 1, the large 3.7 kb transcript appears at 48 h post-infection (Figure 7A) and is inhibited to a large extent but not completely by 400 μM foscarnet (Figure 7B). The small amount of detectable transcript in the presence of foscarnet suggests early-late temporal control. A similar set of blots was hybridized with riboprobe 2. In the absence of foscarnet the 2.5 kb transcript is detected at the earliest time of 24 h and remains detectable up to 144 h post-infection (Figure 7C). As shown in Figure 7D, foscarnet does not affect the 2.5 kb transcript, which indicates its early temporal expression. The 3.1 kb transcript does not appear until 48 h post-infection but is expressed up to 144 h post-infection. This transcript is completely inhibited by foscarnet (Figure 7D), which is consistent with the characteristics of true late temporal expression.

The size of the RT-PCR product obtained using primers flanking UL146 and UL132 (Figure 5) plus the length of the 3.7 kb transcript detected by riboprobes 1, 2, and 3, indicates that this transcript contains more than the UL146, UL147, and UL147A ORFs. Riboprobes 4 and 5 were designed to detect the downstream ORFs UL148 and UL132. In the absence of foscarnet, riboprobe 4 (antisense to both UL148 and UL132) detected 3 smaller transcripts of approximately 2.2 kb, 1.7 kb, and 1.0 kb in size in addition to the larger transcripts detected by riboprobes 1, 2, and 3 (Figure 7E). In Figure 7F the 1.7 kb transcript is unaffected by foscarnet (early transcript), while the 2.2 kb transcript appears to be inhibited by the drug (late transcript). The larger transcripts (3.7, 3.1, and 2.5) show the same temporal expression in the presence of foscarnet as detected by riboprobes 1 and 2. The pattern of transcription detected by riboprobe 5, which is homologous to UL132 was identical to that detected by riboprobe 4 (data not shown). Cellular RNA did not hybridize with any of the riboprobes (Figure 7C and 7E, lanes labeled U).

The northern results reveal that the largest RT-PCR product spanning UL146 through UL132 is derived from the 3.7 kb transcript, because UL146 sequences are not present on any of the other transcripts detected from this region. The size of the 3.7 kb transcript would exclude additional ORFs downstream of UL132 in the absence of splicing. No splicing was detected by sequencing the corresponding RT-PCR product. The 3-prime UL147 ORF sequences are present on the next smaller transcripts (3.1 and 2.5 kb). UL132 sequences are present on all of the transcripts associated with this region, which suggests there may be a common transcriptional 3-prime end. Analysis of the Toledo sequence for polyadenylation signals provides further evidence for 3' co-terminal transcripts, because there is a single consensus sequence at bp 12,563 just downstream of UL132. The next closest signals are 2.8 kb upstream and 2.3 kb downstream of this site, which are not consistent with the size and hybridization patterns of the observed transcripts. HCMV strains Merlin (GenBank Accession # AY446894) and 3157 (GenBank Accession # AY446867) have similar patterns of poly A signals in the homologous region.

## Discussion

This study has focused on a region of the HCMV genome that encodes products potentially very important for the overall pathogenesis of the virus. To our knowledge this is the first comparative study of all of the genomic components of the ORFs from UL146 through UL147A and the first report of multiple overlapping transcripts expressed from this region.

The results show that among clinical HCMV strains there is a gradient of sequence variability that ranges from very high to low beginning with the UL146 ORF and progressing downstream through UL147A. Paradoxically, the hypervariable UL146-UL147A sequences of individual strains were found to be completely stable throughout long term propagation in vitro and in vivo.

The adjacent UL146 and UL147 ORFs have conserved CXC chemokine motifs and are positionally conserved in all clinical strains of HCMV that have been characterized, although they have been deleted from some laboratory strains [5]. They are therefore not essential for virus replication in vitro, but appear to be maintained for infectivity in vivo. The pattern of multiple CXC chemokine homologues is also found in the genomes of other primate CMVs [24,33,34] but not murine CMV (MCMV) [20] perhaps reflecting a divergence in evasion strategies.

The cumulative evidence from this study and others clearly establishes the hypervariability of the UL146 ORF among clinical HCMV strains [2,9,10,22]. These results emphasize that within a defined sequence group, UL146 sequence similarities exist among unrelated clinical strains from widely separated geographic areas, while at the same time there is a high level of UL146 sequence divergence between different groups within individual geographic areas. Despite the hypervariability, all reported UL146 sequences including those in the present study have conserved functional residues associated with CXC chemokines [2,9,10,22]. ELR residues adjacent to the CXC motif are also present in most of the UL146 sequences. ELR-positive CXC chemokines have been reported to induce angiogenesis and vascular remodeling through binding to CXCR2, while ELR-negative chemokines are angiostatic [26,35,36]. The UL146 protein expressed from the Toledo strain induces CXC chemokine functions including neutrophil chemotaxis, calcium flux, and degranulation and binds to CXCR2 [18]. However, the angiogenic activity of UL146 has not been determined. In UL146 Group 5 sequences (Figure 2) the ELR residues are replaced with NGR. The arginine residue that is considered to be absolutely essential for receptor binding [27] is retained, but the potential effect of the NG substitution on chemokine functions is not known. All of the UL147 sequences have DXR residues in the homologous positions of the ELR residues next to the CXC motif. Similar to the UL146 sequence, the arginine residue required for receptor binding is conserved in all UL147 sequences, but no chemokine-related functions have yet been attributed to the UL147 product.

Recently it was reported that ELR-positive CXC chemokine activity is elevated in association with bronchiolitis obliterans syndrome (BOS) [35] in lung transplant recipients. In association with the elevated CXC chemokines there was vascular remodeling of the trachea and aberrant angiogenesis. HCMV infection and disease is a frequent and serious complication for lung transplant recipients. Although HCMV infection was not included in the analysis for the BOS study, these new findings suggest a possible functional link between HCMV chemokine activity and human disease. It will be important for HCMV pathogenesis to determine whether such a link exists and how sequence variability could affect this function.

Further sequence examination of the intergenic region between UL146 and UL147 produced two unexpected findings. First, it was found to be highly variable in both nucleotide length and sequence. This is surprising because in previous analyses of other variant HCMV genes [7](N.S. Lurain, unpublished data), the coding sequences from all unrelated clinical isolates could be amplified using a single set of primer pairs from the non-coding flanking regions, suggesting that the sequences of these flanking regions are generally conserved. The upstream non-coding sequence of UL146 has conserved primer binding sites, but the intergenic region provides no conserved downstream site. A second unexpected finding is the consistent linkage of the highly variable intergenic sequences with specific UL146 ORF sequence groups. The UL147 ORF has lower overall sequence variability than the UL146 ORF. However, phylogenetic analysis based on UL147 sequences shows that the strains cluster in the same groups determined by the UL146 and intergenic sequences. Thus, there is no evidence for recombination between UL146 and UL147.

The start site of the UL147A ORF is invariably only 2 nucleotides downstream of the UL147 stop codon. Despite the highly conserved sequence of the UL147A ORF, this very short 2-basepair sequence between the UL147 and UL147A ORFs strongly suggests linkage to the rest of the region. However, there is no known functional relationship between the predicted products of UL147 and UL147A.

In contrast, the present study established that the sequence groups of the hypervariable UL144 ORF are not linked to the UL146 sequence groups even though UL144 is less than 1 kb upstream of UL146. We have previously shown UL144 to be unlinked to the variable gB gene, which is more than 90 kb upstream [7]. These data along with those reported by others indicate that most of the known hypervariable ORFs are unlinked, which suggests that so far, with the exception of UL146 and UL147, the pattern of known variant genes present in each strain was most likely generated over very long periods of time by recombination events [8,11,15]. The lack of recombination within the region UL146 through UL147A is further supported by the fact that there are HCMV strains from each geographic site that have identical sequences spanning this entire region. This raises the question of how the hypervariability has evolved. We addressed this question first by investigating the possibility of cumulative sequence drift over long-term virus propagation. Serial passage of multiple clinical isolates over several months in cell culture failed to produce even a single nucleotide substitution despite phenotypic changes from cell-associated to cell-free virus. This in vitro approach confirms that long-term cell culture does not add sequence artifacts. However, cell culture lacks components of the immune system that could produce sequence drift through selection of antigenic variants.

The possibility of sequence drift in vivo was addressed by analyzing sequential isolates obtained from transplant recipients over long-term follow-up of several months to several years, a much longer period of follow-up than that of previous studies [10,22]. All isolates from specimens from the same patient including those from different body compartments maintained identical UL146 sequences demonstrating that no sequence drift occurred in vivo. The most convincing evidence that in vivo passage of HCMV strains does not produce sequence drift comes from the data from four matched pairs of transplant recipients with the same donor. All isolates from related patient pairs have identical nucleotide sequences of the UL146-UL147A region, and in the case of CH1 and CH2 the sequence identity was maintained over a period of almost 5 years. Thus, passage of the same strain in different hosts did not select variant UL146 sequences.

Some patients had evidence of infection with more than one strain, for example subjects R1 and NW23 (Table 2). However, the UL146-UL147A sequences of each individual strain remained stable over long-term passage both in vitro and in vivo with no evidence of recombination. We would predict from the observed sequence stability of HCMV strains during long-term passage that even minor sequence differences among isolates from the same patient indicate the presence of multiple strains rather than sequence drift of a single strain. This prediction can be confirmed by analyzing the sequences groups of other unlinked variable genes such as UL144 and gB detected among the same isolates.

The close linkage and sequence stability of the UL146-UL147A ORFs led to the investigation of potential splicing and temporal expression of transcripts from this region. Analysis of RT-PCR products revealed a single large transcript that contained not only the UL146-UL147A ORFs but also the downstream UL148 and UL132 ORFs. The RT-PCR sequences showed no evidence of spliced transcripts. Northern analysis identified a dominant large 3.7 kb transcript that hybridized with riboprobes representing all 5 ORFs, and also identified 5 other transcripts ranging in size from approximately 1.0 to 3.1 kb that hybridized with riboprobes from one or more of the ORFs. UL146 sequences were only detected on the largest transcript (3.7 kb), and UL132 sequences were detected on all transcripts. The transcripts represent different temporal classes as determined by the time of expression post-infection and by the effect of foscarnet on that expression. Based on size and hybridization patterns, UL146 appears to be expressed only from the large 3.7 kb transcript, which has early-late kinetics. UL147 is likely expressed from the 3.1 kb transcript, which has true late kinetics. These results are slightly different from earlier microarray analysis of HCMV transcriptional expression based on the Towne strain [37], which indicated that UL146 (UL152 in Towne) and UL147 have similar early-late kinetics. The discrepancies likely result from the inability of microarray analysis to distinguish overlapping transcripts. Penfold et al. [18] reported that the UL146 protein is expressed with true late kinetics as shown by foscarnet inhibition, but no transcriptional analysis was reported. However, early-late transcriptional expression is compatible with the potential timing of chemokine activity that would likely play a role in pathogenesis after viral replication.

The northern analysis of the UL146-UL132 ORFs shows a transcriptional pattern and complexity similar to that found in other genomic regions of HCMV including the UL93-UL99 ORFs [38,39], which have: 1) overlapping transcripts with different 5-prime termini; 2) co-terminal 3' ends; and 3) different temporal expression of the transcripts. The RT-PCR and northern data show a series of transcripts that all include UL132 sequences at the 3' end but vary in the number of upstream ORFs and differ in their temporal expression. The single poly A signal, which is downstream of the UL132 stop codon, supports the possibility of a common 3'-terminus for all of these transcripts [2,5].

## Conclusion

Despite an extensive characterization of the UL146-UL147A ORFs, we are left with the question of how the UL146 protein with an apparent defined function that is conserved in all HCMV clinical isolates has developed such a highly variable but stable amino acid sequence. The results of our study allow us to rule out several mechanisms that might generate sequence diversity. Sequence drift was eliminated as a mechanism by the long-term in vitro and in vivo passage of a large number of HCMV isolates, which produced no sequence changes in individual strains. A second possible mechanism is selection of immune escape mutants as has been reported for the m157 ORF of MCMV [40]. The in vivo stability of UL146 and UL147 especially in paired transplant recipients, however, argues against a similar selection for HCMV. An immune escape mechanism may exist for HCMV, but it appears not to be based on either of these two genes. Transcriptional splicing is a third potential mechanism for generating sequence diversity, yet is unlikely, because the single UL146 transcript is unspliced.

Finally, interstrain recombination is a common mechanism for generating sequence variability [15], which requires co-infection by genetically different strains within the same cell. A recent study of co-infection of MCMV strains reports evidence for frequent co-infection of the same cell but little evidence of recombination between the strains [41]. An increase in the fitness of an attenuated MCMV strain in the presence of a wild-type strain was shown to be the result of *trans*-complementation rather than recombination. Similarly, the linkage of the UL146-UL147A ORFs and the stability of individual strains in co-infected patients, do not support recombination as a mechanism for generating the sequence diversity observed in this region. This appears to be an unusual finding among HCMV variable ORFs, because the cumulative data for a number of other variable HCMV ORFs [7,11,15] suggests that recombination between them must have occurred over the course of virus evolution leading to the generation of a seemingly unlimited number of distinguishable HCMV strains. A recent report suggests that intrastrain recombination between UL146 and UL147 may be a mechanism for generating the observed variability [34]. However, the absolute sequence stability in vitro and in vivo along with the conserved groups based on UL146 through UL147A sequences from widespread geographic sites do not appear to support this mechanism.

It is very possible that the variability of the UL146 and UL147 ORFs may have evolved in a host-specific manner over a very long period of time, which has been postulated for hypervariable ORFs found in human herpesvirus 8 [42,43]. The fact that the differences among HCMV strains based on UL146-UL147A sequences do not present as random changes but instead occur as defined sets of amino acid substitutions suggests that there may be selection based on virus and/or host functional constraints. Variability of host factors such as CXCR2 or MHC haplotypes may affect the ability of specific virus genotypes to productively infect individual hosts. Although there is no evidence so far that HCMV strains differ in pathogenicity, undoubtedly both host and viral factors are involved in determining the outcome of HCMV infection.

## Methods

### Virus isolates and specimens

HCMV isolates and HCMV-infected specimens (white blood cells and urine) were obtained at two geographically distant medical centers: the VA Medical Center and Oregon Health and Science University, Portland, OR and Rush University Medical Center, Chicago, IL. Samples from specimens obtained for normal patient care including viral isolates, white blood cells, and urine were collected from selected patients over periods of several months to several years as well as from 4 pairs of solid organ transplant recipients (3 kidney, 1 lung) who were infected through organs obtained from a common HCMV-positive donor. The use of these specimens as discarded clinical material was in accordance with federal guidelines, and the study was approved by the Institutional Review Boards at both Rush University Medical Center and the Oregon Health and Science University.

Viable isolates were received as infected human foreskin fibroblast (HFF) monolayers in tube cultures, which were trypsinized and passed to fresh uninfected monolayers. The level of infectivity was increased by repeated rounds of trypsinization and redistribution of infected monolayers as well as by passage of infected cells to new monolayers. The total number of passages required to reach a level of 60 to 80% infectivity was usually in the range of 4 to 6. All of these strains remained cell-associated and stocks were maintained as infected cells.

For stability studies, selected isolates from Portland were passed multiple times beyond the point at which the virus-infected cells began to release extracellular virus. Cell culture supernatants from these isolates were harvested after multiple subsequent passages.

### DNA extraction and PCR amplification

For the Chicago isolates, CMV genomic DNA was extracted from infected HFF monolayers, culture supernatants, or directly from patient specimens using the QIAamp DNA Mini Kit (QIAGEN, Inc., Valencia, CA). The extracted DNA served as the template for PCR amplification using the GenAmp XL kit (Applied Biosystems, Foster City, CA). For the Portland isolates, HCMV DNA was extracted from infected HFF cultures using a Hirt method [44]. Viral DNA from ultracentrifuged urine sediment was extracted by alkaline lysis as previously described, or by SDS-proteinase-phenol extraction [45].

The UL146 ORF was amplified from DNA extracts from the Portland patient specimens using the outer primers 5'-TTACGGAACCGTGTCTGAGT-3' (forward) and 5'-GTTGATGTG ACGACGCACGGCTTGC-3' (reverse). Nested PCR was performed with the inner primers 5'-GAAACCTAATTGACGTGTGATCG-3' (forward) and 5'-AGCCAGCACTTCCTGACGATT GCAG-3' (reverse). The outer primers alone were used to amplify the UL146 ORF from DNA extracted from infected cells in cultures. The amplification protocol for the Portland isolates was 95°C 2 min for 1 cycle, 15 cycles of 94.5°C for 30 s, 54°C for 30 s, 72°C for 1 min followed by 15 cycles of the same temperatures and times except that the 72°C extension was increased by 5 s per cycle.

For specimens from Chicago both the UL146 and UL147 ORFs were amplified as one product using the primers 5'-GATGTGTCATGGACGCAGTT-3' (forward) and 5'-CAGAAG ATGAGGAGCAGGAA-3' (reverse). PCR amplification was carried out using the GeneAmp XL Kit (Applied Biosystems). The amplification protocol was 94°C 1 min for 1 cycle followed by 30 cycles of 94°C 1 min, 60°C for 10 min.

The UL147A ORF was amplified from the Chicago specimens with the same forward primer used for the UL146-UL147 product described above and the reverse primer 5'-CGCT ACCAGCATGACGTCTC-3', which is downstream of the UL147A stop codon. The resulting product contained the UL146, UL147, and UL147A ORFs. The primers used for the Portland specimens for the same region were 5'-GCTTAAGCCAATCGCAGCGAGC-3' and 5'-GTC GCCTCGGTAGCTCAGTAGC-3'. The amplification protocols were the same as described above for the UL146-UL147 products.

The UL144 ORF was amplified in a subset of the Chicago isolates using the forward primer 5'-TCGTATTACAAACCGCGGAGAGGAT-3' and reverse primer 5'-ACTCAGACACG GTTCCGTAA-3'. The conditions for amplification were 94°C for 5 min followed by 30 cycles of 94°C for 1 min, 55°C for 1 min, 72°C for 1 min and ending with a single extension cycle of 72°C for 5 min.

### DNA sequencing

DNA sequencing reactions were performed using the BigDye Terminator Kit version 3.0 or 3.1 (Applied Biosystems). The reactions were analyzed using either an ABI PRISM 3100 Genetic Analyzer (Applied Biosystems) (Chicago) or ABI 377 Automated Sequencer (Portland). UL146 sequencing primers for specimens from Portland were 5'-GAATTGATGTGTCATG GACGCAG-3' (Forward) and 5'-GACAGGTGTCGTACCGAT-3' (Reverse). For specimens from Chicago, the UL146 coding strand was sequenced using the forward PCR primer above. The reverse sequence was obtained using the primer 5'-CCAGCACTTCCTGACGATTG-3'.

Sequencing of UL147 required additional primers. The 5-prime end of the coding sequence of UL147 could not be obtained with a universal primer, because the intergenic region between UL146 and UL147 does not contain conserved primer binding sites. The sequence of this region was obtained using the reverse primers for UL146 and UL147 (above) and one of the following overlapping reverse primers: 5'-ATCTCTGCGAGGATGCTAGT-3' or 5'-TGGCCAGGCACCGAACTCAA-3'. The 3-prime portion of the UL147 ORF plus the entire UL147A ORF was sequenced using the forward primer: 5'-AAGCTGCAATCGTCAGGAAG-3'. The sequence of the non-coding strand of UL147A was obtained with the reverse PCR primer described above. Portland primers for UL147 sequencing were 5'-GCAGGACGAGCGTGAAC AGC-3' and 5'-GTTGATGTGACGACGCACGGCTTGC-3'.

### RT-PCR

RNA was extracted from infected cells using the RNeasy kit (QIAGEN). The extracts were treated with DNase I (Invitrogen). Reverse transcription was performed using the SuperScript First Strand Synthesis System (Invitrogen). cDNA was produced by amplification with the UL146 forward primer and either the UL147 or the UL147A reverse PCR primers. Additional cDNA products were generated using the UL146 forward primer with reverse primers downstream of the UL148 or UL132 ORFs. The UL148 reverse primer sequence is 5'-TC TTGCTATGTCCGCGAACG-3', and the UL132 reverse primer sequence is 5'-AGATCCC GAGTACGACTAGG-3'. Control reactions that did not include the reverse transcription step were amplified in parallel reactions to check for DNA contamination.

All RT-PCR products were sequenced to check for splicing. The primers described above were used for the UL146-UL147A portion of the cDNA products. An additional sequencing primer was used for the UL148 coding strand: 5'-ACGCTCCTCGTCACTTGTGT-3'. Three additional sequencing primers were used for UL132: 5'-TACACCCTGTCACCGAA AGC-3' (forward); 5'-CTGATCGCGGTAGTTTACTC-3' (forward); and 5'-TCACGAACGAC GTGTCCAAG-3' (reverse).

### Northern analysis

Northern analysis using the NorthernMax-Gly kit (Ambion) protocol was performed on the same RNA extracts used for RT-PCR. In addition, RNA was extracted at 24, 48, 72, 96, and 144 h post-infection from cells that were infected with selected HCMV strains and grown in the presence or absence of 400 μM foscarnet. RNA Millennium Markers™ (Ambion) were run on each gel for size determinations. RNA samples were mixed with loading buffer containing glyoxal followed by electrophoresis on 1% agarose gels. RNA was transferred to Brightstar nylon membranes (Ambion). Five different riboprobes were made using cloned templates of cDNA products inserted into the pGem-TA vector (Promega) (Figure 1). Riboprobe 1 contains 216 bp specifically antisense to the UL146 ORF of isolate CH-22 and 87 bp of the upstream UL146 flanking region. Riboprobe 2 is antisense to 1095 bp of the coding sequences containing the complete UL146 and UL147 ORFs from isolate CH-22. Riboprobe 3 is antisense to 177 nucleotides of the UL147 ORF (bases 225 to 402) from isolate CH-19. Riboprobe 4 is antisense to 1990 bp spanning the coding sequences of UL148 and UL132, and riboprobe 5 is antisense to 303 bases of the UL132 ORF. Both riboprobes 4 and 5 were derived from isolate CH-19. A riboprobe was also produced from the Millennium Marker™ Probe Template (Ambion) for detection of the RNA Millennium Markers™. The recombinant plasmids were linearized and RNA representing the non-coding strand was generated using either T7 or SP6 RNA polymerase and labeled by incorporation of digoxigenin-dUTP (Roche Applied Science). Hybridization of RNA blots was carried out at 68°C overnight. The blots were visualized by addition of anti-digoxigenin antibody labeled with alkaline phosphatase followed by addition of the substrate 5-bromo-4-chloro-3-indolyl phosphate (BCIP) and nitroblue tetrazolium (NBT).

### Phylogenetic analysis

The amino acid sequences of the UL146 and UL147 sequences were aligned using Align Plus 5 version 5.11 (Scientific and Educational Software). Gaps were removed using the Gapstrip tool from the Los Alamos National Laboratory . Phylogenetic analysis was performed on the processed sequences using program modules from the Phylip program package version 3.6a2, obtained from J. Felsenstein, University of Washington, Seattle, WA [46]. The SEQBOOT module was used to generate 100 bootstrap data sets. Genetic distances were calculated from the bootstrap data using PROTDIST, and the resulting values were used to generate phylogenetic trees using NEIGHBOR. A consensus tree was computed by CONSENSE. The depiction of the tree was produced using TreeView [47]. For the UL146-147 intergenic region a dendrogram was generated from the DNA sequences using Align Plus 5 version 5.11 (Scientific and Educational Software).

### Nucleotide sequence accession numbers

The accession numbers for 18 of the UL146 sequences analyzed in Figure 2 are: DQ115708 through DQ115725. The nucleotide accession numbers for the UL146 through UL147A sequences of the 30 clinical strains analyzed in Figures 3 and 4 are: DQ115727 through DQ115756.

## Competing interests

The author(s) declare that they have no competing interests.

## Authors' contributions

NSL conceived the study, analyzed the data from the Chicago site, and drafted the manuscript. SC contributed to the design of the study, analyzed the data from the Portland site, and helped to draft the manuscript. AMF performed the sequencing and transcriptional analysis at the Chicago site. HML performed the sequencing and participated in the analysis at the Portland site. DDH provided essential expertise for the phylogenetic analyses. SMB and ERG provided clinical specimens and clinical data for the Chicago site. CFW provided critical intellectual input and interpretation. SPK provided essential input for the transcriptional analysis.
